# Epileptic seizures during Non-Ketotic Hyperglycemia (NKH) in French Guiana: A retrospective study

**DOI:** 10.3389/fendo.2022.946642

**Published:** 2022-08-18

**Authors:** Dimitri Baltyde, Bertrand De Toffol, Mathieu Nacher, Nadia Sabbah

**Affiliations:** ^1^ Department of Endocrinology and Metabolic Diseases, Cayenne Hospital Center, Cayenne, French Guiana; ^2^ Clinical Investigation Center Antilles French Guiana (CIC INSERM 1424) Cayenne Hospital Center, Cayenne, French Guiana; ^3^ Department of Neurology, Cayenne Hospital Center, Cayenne, French Guiana

**Keywords:** diabetes, non-ketotic hyperglycemia, epileptic seizures, neuroendocrine entity, French Guiana

## Abstract

**Introduction:**

Epileptic seizures during non-ketotic hyperglycemia (NKH) represent a rare complication of uncontrolled diabetes mellitus. The definition associates a blood sugar level > 200mg/dL (11mmol/L), hyperosmolality, absence of ketosis, dehydration and seizure control after normalization of blood sugar levels.

**Material and methods:**

This retrospective observational study included patients hospitalized for epileptic seizures and NKH in the Cayenne Hospital Center between January 2010 and June 2020. The clinical, biological, and radiological results were collected.

**Results:**

18 out of 228 (7.9%) patients with both diabetes and epileptic seizures had NKH. The mean age of the 12 women and 6 men was 64.8 years. In 8 patients, brain imaging did not show acute lesions and the seizures disappeared with control of hyperglycemia by hydration and insulin. In 6 patients, the seizures revealed a stroke, hemorrhagic in 4 cases, ischemic in 2 cases. 4 patients had a seizure in a context of known vascular epilepsy. The epileptic seizures were mainly focal seizures with motor symptoms that could be repeated, focal to bilateral tonic-clonic or focal status.

**Conclusion:**

Seizures in NKH are symptomatic of an acute brain lesion or vascular epilepsy more than 1 in 2 times. However, isolated NKH can cause seizures with a suggestive brain MRI.

## Introduction

Hyperglycemic seizures represent a clinical condition with high blood glucose levels, normal or increased serum osmolality, negative urine ketone bodies and dehydration. Non-ketotic hyperglycemia (NKH) is a rare complication of diabetes mellitus where seizures spontaneously resolve with glycemic control. The definition associates a blood sugar level > 200mg/dL (11mmol/L), hyperosmolality, absence of ketosis, dehydratation and seizure control after normalization of blood sugar levels.

The first review of the literature on the occasion of the report of 7 observations was published in 1965 (1). Since then, small series of patients(Wang 2017, N=13) (Tiamkao 2003, N=21) (Lammouchi 2004, N=22) (2–4) and many isolated clinical cases have been reported. The most frequently observed epileptic seizures are repeated focal motor seizures or focal motor status of different types. However, the observable epileptic symptoms vary widely: language arrest (5), aphasia (6)aphasic status (7) post-ictal blindness (8), tonic clonic generalized seizures (2, 4, 9). Specific MRI(Magnetic resonance imaging) aspects have recently been described (10–12) as subcortical hypointensity in Flair with low signal on apparent diffusion coefficient (ADC). These abnormalities could be distinguished from transient abnormalities related to epileptic seizures (12). The pathophysiology of epileptic seizures in NKH is not known, an old hypothesis involves a decrease in the levels of GABA(gamma-aminobutyric acid), an inhibitory neurotransmitter, due to metabolic disorders (13).

The control of the seizures with symptomatic treatment of hyperglycemia and the reversibility of the radiological abnormalities suggest an acute transient non-lesional symptomatic disorder. Antiepileptic drugs are ineffective or even deleterious (14).

However, hyperglycemia in the setting of known or unknown diabetes is also a risk factor for ischemic stroke (15). Hyperglycemia is also frequently observed in the acute phase of hemorrhagic stroke (16). In addition, seizures are observed in the initial phase of a stroke in 4.3-6.2% of ischemic strokes and in 10.7-15.6% of hemorrhagic strokes (17).

The aim of our retrospective study was to describe the clinical, biological and radiological aspects of patients with epileptic seizures associated with NKH at Cayenne Hospital in French Guiana. In French Guiana, the prevalence of diabetes is double that of mainland France and uncontrolled diabetes is frequent (18).

## Material and methods

This single-center retrospective study was conducted at Cayenne Hospital in French Guiana. Records of patients aged >18 years hospitalized between January 2010 and June 2020 with coding type 1 or type 2 diabetes and seizure or epilepsy or status epilepticus were extracted, and then records with NKH were selected. We reviewed the emergency and hospital records and selected the files of patients corresponding to the definition of hyperglycemia without ketosis, including a blood sugar level > 200mg/dL (11 mmol/L)(Even if there is not really a definite threshold, it is accepted that hyperglycemia is defined by a blood sugar level above 200 mg/dl* * (19, 20), absence of ketosis on a urine dipstick, i.e., less than 2 crosses, or capillary ketonemia less than 0.5 mM/L. Definition of vascular epilepsy is: According to the revised classification of the International League Against Epilepsy (ILAE) a late-onset seizure (i.e at least two weeks after the stroke) in the context of stroke defines vascular epilepsy, making it no longer necessary to wait for a second seizure. For each patient, age, medical history, blood glucose and glycated hemoglobin values, type of seizure, clinical examination, brain imaging data, and length of stay were collected. All patients had had a brain CT scan. EEG was not performed in few cases of generalized status epilepticus because the diagnosis is clinical

### Ethical aspects

The General Data Protection Regulation procedures included recording the study protocol on the health data hub platform under the project title EPIDIAB line 3243. We provided a declaration to the CNIL(The National Commission for Information Technology and Civil Liberties) with the EPIDIAB project title under registration number 2215827. A written information note was sent to all patients in order to confirm their non-objection, as required by French law.

## Results

**Figure 1:**
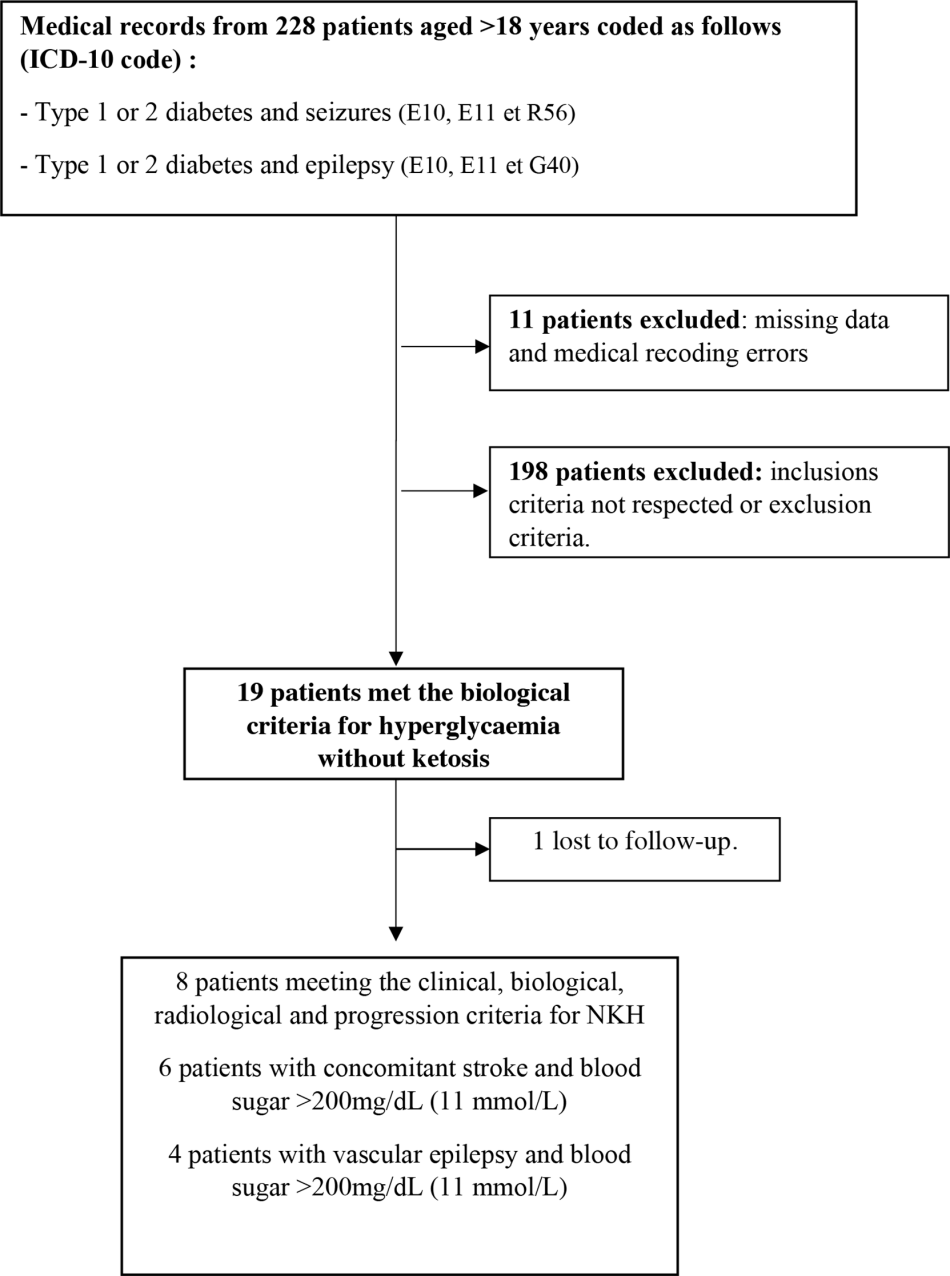
Flow-chart.

The flow chart is presented in **Figure 1**. Among 228 diabetic patients with epileptic seizures, 18 had NKH (7.9%). The **Table 1** summarizes the data collected from the 18 patients. Two different situations can be distinguished.

**Table 1:** Clinical, biological, radiological data and length of stay of adult patients with epileptic seizures during non-ketotic hyperglycemia, 2010-2020, Cayenne hospital, French Guiana.

Case no./SexAge(years)	History	Neurological symptoms	Blood sugar (mmol/dL)	HbA1c %(mmol/mol)	Brain imaging	Length of stay
**1/** **F** **43**	IRD Dyslipidaemia Retinopathy Neuropathy Hypertension BP 205/105	3 GTCSs Left hemiparesis	23.7	11 (97)	-CT Ancient Left lacunar infarct	13
**2/** **F** **52**	Discovery diabetes Hypertension BP 165/80	3 focal motor seizures to bilateral tonic clonic	33.0	15 (140)	-CT Normal	4
**3/** **F** **70**	Discovery diabetes Hypertension BP 240/130	1 focal motor seizure visual and auditive Hallucinations Right hemiparesis	24.1	11.2 (99)	-MRI Hyposignal ADC and Flair in the occipital region	15
**4/** **F** **70**	NIRD Hypertension Ischemic Cardiopathy BP 220/130	Status epilepticus	30.9	12.2 (110)	-CT Leukoaraiosis	7
**5/** **M** **84**	NIRD Hypertension BP 190/110	focal motor seizure to bilateral tonic clonic	13.0	NA	-CT Leukoaraiosis	1
**6/** **M** **73**	NIRD hypertension CRF BP 158/80	4 GTCSs	28.0	11.6 (103)	-MRI Leukoaraiosis Hyposignal ADC and Flair in the right frontal region	10
**7/** **M** **71**	NIRD -hypertension CRF	Aphasia with right facial myoclonic jerks	38.6	11.9 (107)	-CT Normal	3 Self-discharged against medical advice
**8/** **F** **49**	IRD	Myoclonic jerks of the upper limbs and face	43.0	14.1 (131)	-CT Normal	1
**9/** **F** **49**	NIRD Hypertension Retinopathy Dyslipidaemia 3 strokes	Aphasia Right hemiparesis Left facial paralysis Right third nerve palsy 1 GTCS	13	NA	- Ancient left frontal ischemic stroke -2 left frontal lobe hematomas	46
**10/** **F** **74**	Hypertension IRD	Status epilepticus Right hemiparesis	21.10	NA	-Left hemisphere hematoma	5
**11/** **M** **62**	NIRD Hypertension -Hypertensive cardiopathy Dyslipidaemia. 2 strokes	1 GTCS	12.04	NA	-Right fronto-parietotemporal ischemic stroke	2
**12/** **F** **78**	NIRD Hypertension	2 focal occipital seizures	11.66	NA	-Left parietal hematoma	10
**13/** **F** **51**	Discovery diabetes Hypertension OSA 2 haemorrhagic strokes	Dysarthria 5 GTCSs	17.60	12.8 (116)	-Left frontal hematoma -Right parietal hematoma -Subcortical microbleeds	13
**14/** **F** **66**	NIRD Hypertension Ischemic stroke Ischemic heart disease	2 GTCSs Left hemiplegia	22	8.8 (73)	-Right ischemic stroke	17
**15/** **F** **62**	IRD Retinopathy Nephropathy Neuropathy Haemorrhagic stroke Vascular Epilepsy	5 GTCSs Right facial paralysis + head and eye deviation	19.7	14.5 (135)	-Ancient stroke	14
**16/** **M** **69**	NIRD Hypertension -Cerebellar stroke Two seizures before	Aphasia Focal to bilateral tonic clonic seizure	22.2	8 (64)	-Left cavernoma	10
**17/** **F** **76**	NIRD Hypertension -Right frontal Ischemic stroke Dyslipidemia Vascular epilepsy	Status epilepticus	15.6	6.5 (47.5)	-Ancient stroke cortical subcortical atrophy	47
**18/** **M** **66**	NIRD Neuropathy	3 GTCSs	20.5	14.1 (131)	-Ancient stroke (not known) leukoaraiosis	7

F, female; M, male; IRD, insulin-requiring diabetes; NIRD, non-insulin-requiring diabetes; NA, not available; GTCS, generalized tonic-clonic seizure; d, days; OSA, obstructive sleep apnoea; CRF, chronic renal failure; BP, Blood Pression.

In the first group 5 patients had an oral anti-diabetic treatment and 2 had insulin glargine with oral agent, 1 was new case of diabetes. In the second group 5 had an oral anti-diabetic treatment, 3 patients had insulin glargine and oral agent and one only basal bolus, and one was a new case of diabetes.

**Figure 2:**
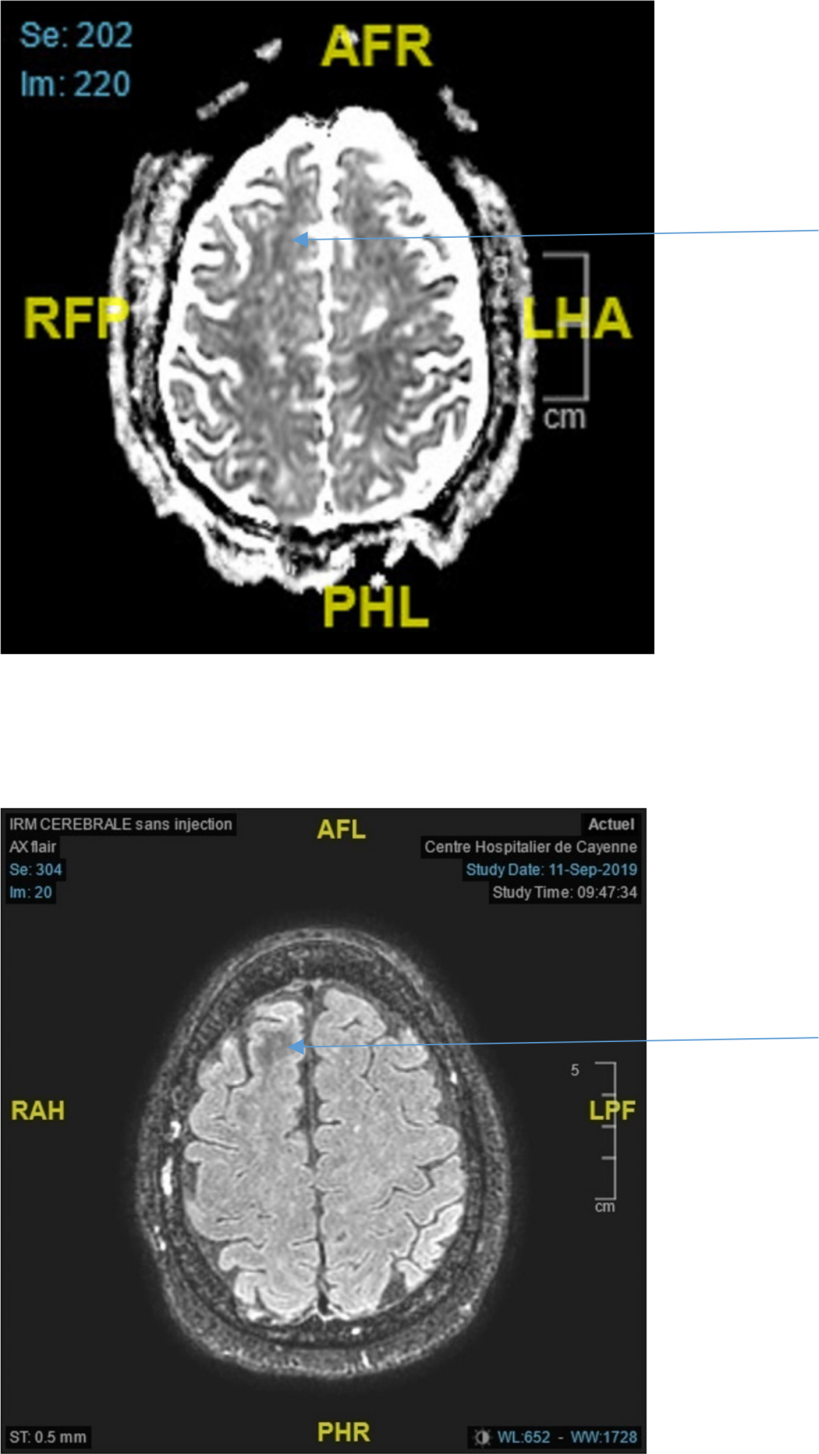
Top ADC decrease in the right frontal lobe (arrow) Bottom Axial Flair showing white matter hyposignal in the right frontal lobe (arrow).

For a first group of subjects (patients 1 to 8) the brain imaging did not show acute or ancient brain lesions. Seizures were focal to bilateral tonic-clonic in 5 out of 8 cases: 4 focal motor, one focal cognitive. One patient had generalized status epilepticus (case 4). One patient experienced four generalized tonic-clonic seizures (case 6) while another patient experienced three generalized tonic-clonic seizures (case 1). EEGs (electroencephalogram) performed in all patients were normal. Brain MRI was performed in 2 patients (patients 3 and 6) and showed subcortical hyposignals in Flair with ADC decrease (**Figure 2**). In 2 patients (patients 2 and 3), seizures in relation to hyperglycemia revealed previously unknown diabetes. The mean age of the five women and three men was 64 years (range, 43-84 years). On admission, the mean blood glucose concentration was 29.3 mmol/L (range, 13-43 mmol/L), while the mean osmolarity and serum sodium, urea, bicarbonate, and glycated hemoglobin levels were, respectively, 301.5 mOsmol/L (range, 293.3-316 mOsmol/L) and 133.3 mmol/L (range, 127-139 mmol/L), 6.83 mmol/L, (range, 4.6-13.8 mmol/L), 24.5 mmol/L (range, 20.2-31 mmol/L), and 12.42% (112mmol/mol) (range, 11%-15% (97mmol/mol-140mmol/mol)). All patients were rehydrated with isotonic saline associated with venous insulin therapy. Seizures were rapidly controlled with symptomatic treatment of hyperglycemia. The mean length of stay of patients in this group was 7 days (range, 1-15 days). The rehydration rates were different. Patient no. 8 received the highest rehydration volume of 3 L/24 hours with venous insulin therapy at the highest rate of 7 UI/h, and her hospital stay was the shortest (1 day). Patient No. 7 had a stay of 3 days due to discharge against medical advice.

For a second group of subjects (patients 9 to 18), the seizures were contemporary with an acute stroke in 6 (4 hematoma, 2 ischemia) or related to a vascular epilepsy in 4 with ancient epileptogenic vascular lesions on brain imaging. The seizures were generalized tonic in 7 patients. One patient had repeated focal occipital seizures and two patients had a generalized convulsive status. Seven out of 10 patients underwent electroencephalograms, two were normal, 3 showed slowing of background activity and 2 showed PLED’s (periodic lateralized epileptiform discharges). The brain imaging results revealed 4 hemorrhagic strokes and 2 ischemic strokes, one cavernoma, and 3 ancient strokes. The mean age of the seven women and three men was 65.2 years. The mean blood sugar concentration, glycated hemoglobin levels and corrected osmolarity of this group were, respectively, 319 mg/dL (17.54 mmol/L), 10,8% (95mmol/mol (6,5-14,5% (48-135mmol/mol)) and 295.8 mOsmol/L. The average length of stay was 13.9 days. All patients were rehydrated; half of the patients received insulin treatment.

## Discussion

The occurrence of seizures in a diabetic patient with NKH corresponds to 2 distinct situations: in the majority of cases, the seizures reveal an ischemic or hemorrhagic stroke or testify to a pre-existing vascular epilepsy. However, it can also be a transient metabolic phenomenon that is reversible with rehydration and insulin therapy. Brain imaging makes the diagnosis.

Seizures in NKH in the absence of brain damage represent 3.5% of our sample of diabetic patients with seizures. This is a rare situation. This percentage is much lower than those reported in in the older literature before the existence of modern brain imaging estimating NKH to represent 15-40% (1) or 25% (21) of seizures. The 8 patients with a reversible transient metabolic disorder after rehydration and insulin therapy correspond to the data in the literature (2, 4, 14): average age over 60 years, predominance of repeated focal motor seizures or in the form of focal status, rapid control of seizures with normalization of blood glucose (9). The mean values of blood glucose and osmolality were respectively 29.3 and 301 in our series versus 28.7 and 300 (4); 28.3 and 308 (2); 32.6 and 302 (3). Diabetes was in all our cases poorly controlled (HbA1 12.4% (112mmol/mol)). In 2007, Huang et al. established that diabetic patients with glycated hemoglobin values greater than 9% were at greater risk of having a seizure. Four studies investigating this neuroendocrine entity have measured this parameter. These are case reports of single patients, or series of 2 and 3 patients (19, 22). The 7 glycated hemoglobin values reported were 9.4% (79mmol/mol) and 10.5% (91mmol/mol) for the reports; 13.8% (127mmol/mol) and 14.4% (134mmol/mol) for the series of 2 patients; and 14.7% (137mmol/mol), 10.5% (91mmol/mol), and 11.9% (107mmol/mol) for the series of 3 patients.

The discovery of diabetes at the time of the seizures was observed in 2 patients/8 in our series, for 11/22 (4) and 15/21 (3). In our study the mean serum sodium level was 133mmol/L, so it did not participate in the epileptic seizure during the hyperglycemia, knowing that the cut off value responsible for epilepsy in hyponatremia is 115mmo/L (23).

In both cases where MRI was performed it showed Flair hyposignals of white matter related to the region involved in seizures (12) associated with ADC decrease. A recent review (10) collated the abnormalities observed on MRI in 30 NKH patients from 5 publications (19, 24–26): a T2/Flair hyposignal of white matter is observed in 28/30 patients. For Urbach, 2020, T2/Flair hyposignal with ADC decrease in a focal seizure context should raise the possibility of NKH (12). The ADC decrease would indicate the presence of cytotoxic edema. The transient deposition of free radicals and/or iron because of excitotoxic axonal damage during hyperglycemia-induced seizures and intracellular dehydration in glial and supporting tissues are postulated mechanisms for subcortical altered signal intensity (27). Epileptic seizures result in T2 Flair or diffusion hypersignals depending on the time course and irrespective of their cause. In case of Flair hyposignal there is no ADC decrease in an epileptic context without hyperglycemia (28).

For patients with NKH, the main explanation is an increased metabolism of GABA which is the major inhibitory neurotransmitter in the central nervous system. Cellular dehydration caused by increased intra-extraneuronal osmolality and hypoxia depresses the Krebs cycle. To compensate for the consequent deficit in brain glucose, GABA is metabolized to succinic acid, fulfilling 40% of the nervous system’s energy requirements (11). This former explanation does not adequately account for the abnormalities seen on MRI and the small number of NKH patients who have seizures.

In the most common situation (10 patients/18) seizures are associated with an acute vascular brain injury or are related to vascular epilepsy. In the series of 21 patients by Singh and Strobos, 1980, at least 13 patients had a documented brain lesion and the metabolic disorder was considered as the triggering factor of the seizures but not as its sole cause (29).

The existence of diabetes and hyperglycemia are two independent factors for poor prognosis of intracerebral hematomas (30). In addition, the incidence of late-onset post-stroke seizures has been estimated at 8.2% for all types of strokes (31) while the rate at 5 years has been 9.5% for ischemic strokes. In hemorrhagic strokes, the rate is slightly higher (11.8%) Haapaniemi 2014 (32). Hyperglycemia in this context is a factor favoring epileptic seizures. The glycemic control of the previous months is also an important factor to take into account, with an increased risk in patients with diabetes of having epileptic seizures in NKH. It should be noted that we do not find epileptic seizures in ketotic hyperglycemia that must be extremely rare (29).

## Conclusion

This retrospective study shows that epileptic seizures in NKH may correspond to a rare and specific neuro-endocrine entity, reversible within 24 hours with rehydration and insulin therapy and not requiring antiepileptic treatment. Brain MRI shows abnormalities suggestive of the diagnosis. However, in the most common situation, epileptic seizures in NKH are related to acute or ancient brain damage observed on the brain MRI and in a context of glycemic imbalance.

## Data availability statement

The original contributions presented in the study are included in the article/Supplementary Materials. Further inquiries can be directed to the corresponding author.

## Ethics statement

The studies involving human participants were reviewed and approved by 2215827. Written informed consent for participation was not required for this study in accordance with the national legislation and the institutional requirements.

## Author contributions

Conceptualization: DB, BD, MN, and NS. Data curation: DB, MN, and NS. Formal analysis: DB, BD, MN, and NS. Investigation: DB, BD, MN, and NS. Resources: DB, BD, MN, and NS. Supervision: MN and NS. Validation: DB, BD, MN, and NS. Visualization: DB, BD, MN, and NS. Manuscript writing: DB, BD, MN, and NS.

## Acknowledgments

This work was realized in close collaboration with the Centre d’Investigation Clinique Antilles Guyane, Inserm CIC 1424.

## Conflict of interest

The authors declare that the research was conducted in the absence of any commercial or financial relationships that could be construed as a potential conflict of interest.

## Publisher’s note

All claims expressed in this article are solely those of the authors and do not necessarily represent those of their affiliated organizations, or those of the publisher, the editors and the reviewers. Any product that may be evaluated in this article, or claim that may be made by its manufacturer, is not guaranteed or endorsed by the publisher.

## Abbreviations

NKH, Non ketotic hyperglycemia; MRI, Magnetic resonance imaging; GABA, gamma-aminobutyric acid; ADC, Apparent Diffusion Coefficient; EEG, electroencephalogram CNIL, The National Commission for Information Technology and Civil Liberties; PLED’s, periodic lateralized epileptiform discharges.
